# Plasma TNFSF10 levels associated with acamprosate treatment response in patients with alcohol use disorder

**DOI:** 10.3389/fphar.2022.986238

**Published:** 2022-09-01

**Authors:** Ming-Fen Ho, Cheng Zhang, Irene Moon, Brandon J. Coombes, Joanna Biernacka, Michelle Skime, Doo-Sup Choi, Paul E. Croarkin, Mark A. Frye, Quyen Ngo, Cedric Skillon, Tyler S. Oesterle, Victor M. Karpyak, Hu Li, Richard M. Weinshilboum

**Affiliations:** ^1^ Department of Molecular Pharmacology and Experimental Therapeutics, Mayo Clinic, Rochester, MN, United States; ^2^ Division of Computational Biology, Quantitative Health Sciences, Rochester, MN, United States; ^3^ Department of Psychiatry and Psychology, Mayo Clinic, Rochester, MN, United States; ^4^ Hazelden Betty Ford Foundation, Mayo Clinic, Center City, MN, United States

**Keywords:** alcohol use disorder, acamprosate, TNFSF10, proteomics, alcohol craving, treatment outcomes

## Abstract

Acamprosate is an anti-craving drug used in alcohol use disorder (AUD) pharmacotherapy. However, only a subset of patients achieves optimal treatment outcomes. The identification of predictive biomarkers of acamprosate treatment response in patients with AUD would be a substantial advance in addiction medicine. We designed this study to use proteomics data as a quantitative biological trait as a step toward identifying inflammatory modulators that might be associated with acamprosate treatment outcomes. The NIAAA-funded Mayo Clinic Center for the Individualized Treatment of Alcoholism study had previously recruited 442 AUD patients who received 3 months of acamprosate treatment. However, only 267 subjects returned for the 3-month follow-up visit and, as a result, had treatment outcome information available. Baseline alcohol craving intensity was the most significant predictor of acamprosate treatment outcomes. We performed plasma proteomics using the Olink target 96 inflammation panel and identified that baseline plasma TNF superfamily member 10 (TNFSF10) concentration was associated with alcohol craving intensity and variation in acamprosate treatment outcomes among AUD patients. We also performed RNA sequencing using baseline peripheral blood mononuclear cells from AUD patients with known acamprosate treatment outcomes which revealed that inflammation-related pathways were highly associated with relapse to alcohol use during the 3 months of acamprosate treatment. These observations represent an important step toward advancing our understanding of the pathophysiology of AUD and molecular mechanisms associated with acamprosate treatment response. In conclusion, applying omics-based approaches may be a practical approach for identifying biologic markers that could potentially predict alcohol craving intensity and acamprosate treatment response.

## Introduction

Acamprosate has been approved in the United States by the FDA for the treatment of alcohol use disorder (AUD). However, only a subset of patients achieves optimal treatment outcomes (Ho et al., 2022a). Acamprosate is not protein-bound; nor is it metabolized; and it is excreted unchanged in the urine (Más-Serrano et al., 2000; Kalk and Lingford-Hughes, 2014). It has been reported that acamprosate helps balance disrupted GABAergic and glutamatergic neurotransmission by decreasing over-excitation induced by alcohol (Mason and Heyser, 2010; Witkiewitz et al., 2012). However, the molecular mechanism(s) of action of acamprosate as a treatment for AUD remain unclear. While the identification of clinical predictors of pharmacological outcomes in response to the drug therapy of AUD patients has not yet been successful (Verheul et al., 1999; Verheul et al., 2005), it would be a significant achievement for addiction medicine, if we could identify potential biomarkers that might be associated with variation in acamprosate treatment outcomes in patients with AUD. A major challenge facing neuropsychopharmacology is the fact that, in psychiatry, we lack “objective biochemical measures” as indicators to assist with diagnosing or evaluating treatment response, and the fact that many clinical phenotypes are not yet closely linked to underlying biology. Therefore, the application of omics-based approaches might represent a useful research tool for discovery of the underlying biology and the identification of novel therapeutic targets.

We previously conducted an acamprosate clinical trial to identify potential biomarkers associated with acamprosate treatment response using metabolomics and genomics (Hinton et al., 2017; Ho et al., 2020; Biernacka et al., 2021). Those AUD patients were treated with acamprosate for 3 months in community-based treatment programs (Karpyak et al., 2014; Karpyak et al., 2019). The primary outcome was abstinence, but we also collected comprehensive clinical information for this study cohort before and after 3 months of acamprosate treatment (Karpyak et al., 2019; Biernacka et al., 2021). Our previous study suggested that genetic variants in the *TSPAN5* gene were associated with acamprosate treatment response (Ho et al., 2020). We found that acamprosate treatment downregulated *TSPAN5* expression which, in turn, decreased concentrations of kynurenine, a major metabolite of tryptophan that plays a role in neuroinflammation (Ho et al., 2020). Knockdown of TSPAN5 also influenced the expression of genes associated with interferon signaling pathways (Ho et al., 2020). In addition, acamprosate has neuroprotective effects by enhancing stability of the blood–brain barrier, reduction of oxidative stress and cerebral leukocyte infiltration (Doeppner et al., 2015). As a result of a growing body of evidence that long-term heavy alcohol consumption could activate immune responses and inflammation (Lowe et al., 2020; Karoly et al., 2021), the present study was designed to explore inflammatory modulators which might be associated with acamprosate treatment outcomes.

In the present study, we performed proteomic assays using the Olink target 96 inflammation panel to identify inflammatory markers which might be associated with acamprosate treatment outcomes and alcohol craving in patients with AUD. We hypothesized that baseline proteomic profiles with a focus on inflammatory markers might differ between patients who maintained sobriety and those who relapsed, and that those differences might provide insight into mechanisms involved in variation in drug response phenotypes (Nam et al., 2015; Frye et al., 2016a; Frye et al., 2016b; Hinton et al., 2017). We also explored differences in proteomic profiles between men and women, since sex differences play a role in AUD pathophysiology (Mason and Lehert, 2012; Karpyak et al., 2019) and may also play a role in response to AUD pharmacotherapy. Finally, we set out to identify the molecular mechanism(s) underlying acamprosate treatment response. The findings described subsequently could serve as an essential step in advancing our understanding of the pathophysiology of AUD and mechanisms of drug action responsible for variation in acamprosate response in patients with AUD.

## Materials and methods

### Study subjects and clinical assessments

The NIAAA-funded Mayo Clinic Center for the Individualized Treatment of Alcoholism study had previously recruited 442 AUD patients who received 3 months of acamprosate therapy (The ClinicalTrials.gov Identifier: NCT00662571). However, only 267 subjects returned for the 3-months follow-up visit and, as a result, had treatment outcome information available. Study subject inclusion criteria include: 1) Age 18 to 70; DSM-5 (Doeppner et al., 2015) diagnosis of AUD determined by The Psychiatric Research Interview for Substance and Mental Disorders (PRISM); 2) Completion of alcohol detoxification (The Clinical Institute Withdrawal Assessment score <5) and no alcohol for at least 7 days (but no more than 21 days); 3) Ability to provide informed consent; and 4) Willingness to use the study medications for 3 months and attend follow-up visits. Exclusion Criteria include: 1) Hypersensitivity or allergy to acamprosate; 2) Renal impairment (creatinine level >1.5 mg/dl); 3) Diagnosis of advanced liver disease indicated in the medical record or by a Model For End-Stage Liver disease (MELD) score of above 10; 4) Women who are pregnant, breastfeeding, or planning to become pregnant during the next year; 5) Primary diagnosis of substance use disorder other than alcohol as determined by PRISM; 6) Current use of benzodiazepines, opioids or any other addictive medications; and 7) Active suicidal ideation or any unstable medical or psychiatric condition as determined by responses to PRISM or by the primary care physicians. Baseline clinical information and biological samples were collected after enrollment and prior to acamprosate treatment.

This study (IRB number: 07-007204) was conducted under protocols reviewed and approved by the Mayo Clinic Institutional Review Board. Confidentiality was maintained for all study participants. We collected clinical data, including the Patient Health Questionnaire 9 (PHQ-9), Generalized Anxiety Disorder Screener (GAD-7), Penn Alcohol Craving Scale (PACS) and psychiatric comorbidities. The primary study outcome was abstinence as determined by timeline followback (TLFB) self-reports (Sobell et al., 1996). Relapse (*n* = 110) was defined as taking a drink during 3 months of acamprosate treatment, while non-relapse (n = 157) was defined as remaining abstinent during 3 months of acamprosate treatment. Heavy drinking was defined as four or more standard drinks per day for a woman and five or more standard drinks per day for a man based on the Dietary Guidelines for Americans 2015–2020 recommendations (https://www.niaaa.nih.gov/alcohol-health/overview-alcohol-consumption/moderate-binge-drinking). We also collected alcohol use information during the 90 days preceding study enrollment using the TLFB self-reports.

### Olink target 96 inflammation panel

Plasma proteomics were measured using Olink proximity extension immunoassays. The Olink inflammation panel includes 96 inflammatory markers (Supplementary Table S1). We assayed all ten plates in a single batch. Data were normalized to standard EDTA plasma controls to produce relative protein abundance information. Analyses reported subsequently excluded proteins with >25% of samples below the limit of detection (Supplementary Table S1). Data are expressed as normalized protein expression (NPX) in arbitrary units on a log2 scale. NPX reflects protein concentration. NPX does not reflect the exact protein concentrations in the samples, however, a high NPX value corresponds to a high protein concentration.

### AUD patient-derived lymphoblastoid cell line model system and drug treatment

**FIGURE 1:**
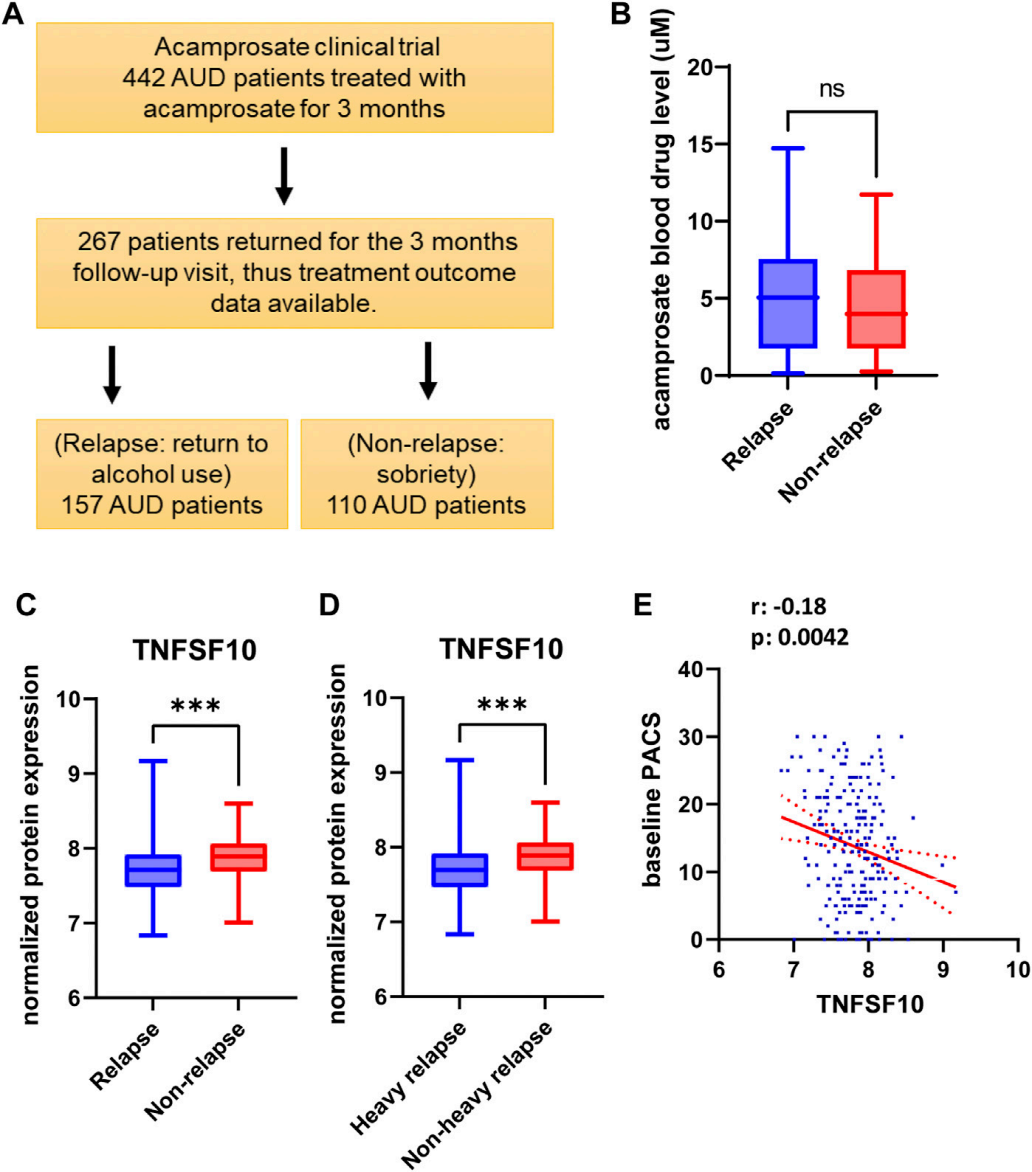
**(A)** Study design and sample numbers for the acamprosate clinical trial. **(B)** Blood concentrations of acamprosate in relapse and non-relapse groups. **(C)** Lower baseline plasma TNFSF10 concentrations were observed in AUD patients who relapsed during 3 months of acamprosate treatment (*t* = 3.658, df = 243, ***p:0.0003). Relapse was defined as taking one drink during 3 months of acamprosate treatment, while non-relapse was defined as remaining abstinent during 3 months of acamprosate treatment. **(D)** Lower baseline plasma TNFSF10 concentrations were also observed in AUD patients who relapsed to “heavy drinking” during 3 months of acamprosate treatment. treatment (*t* = 3.590, df = 223, ***p:0.001). **(E)** Baseline plasma TNFSF10 concentrations were negatively correlated with baseline PACS.

AUD patient-derived lymphoblastoid cell lines (LCLs) were cultured in RPMI 1640 media (Cellgro, Manassas, VA, United States) supplemented with 15% FBS (Atlanta Biologicals, Flowery Branch, GA, United States). Charcoal stripped FBS (10%) was used during drug treatment. Cells were seeded in T75 flasks and were treated with ethanol (EtOH: 25 mM), a concentration considered physiologically relevant for EtOH use, with 25 mM EtOH being slightly higher than the 0.08% blood alcohol concentration that is often used as a measure of intoxication (Lira et al., 2020). The concentration of acamprosate (5 µM, Sigma, A6981) used to perform those experiments was selected to fall within the range of blood concentrations observed during clinical therapy (Mason et al., 2002) (see Figure 1B). Cells were cultured with vehicle, EtOH and acamprosate for 7 days and the medium was changed every day.

### Enzyme linked immunosorbent assay (ELISA) for TNF superfamily member 10

We measured TNFSF10 levels in cell lysates of LCLs derived from AUD patients. We used protein (2.5mg) to measure TNFSF10 concentration using a TRIAL (TNFSF10) human ELISA kit (Cat #BMS 2004, Thermo Fisher Scientific) following the manufacturer’s instructions. We performed ELISA for TNFSF10 in triplicate and read absorbances on a microplate reader at 450 nm (Tecan, Infinite M1000 Pro).

### RNA sequencing and data analysis

Peripheral blood mononuclear cells (PBMCs) samples were isolated from 15 ml of whole blood before acamprosate treatment using Ficoll density gradient centrifugation. We lysed the cells in Trizol and extracted total RNA using the RNeasy mini kit (Qiagen, Valencia, CA, United States). The RNA integrity numbers (RIN) were between 8.5 and 9.2 for the PBMC samples (relapse: n = 27, non-relapse: n = 26). RNA-seq experiments were conducted by GENEWIZ using an Illumina HiSeq 4000 with eight samples in each lane using 100bp paired end index reads. Fastq files containing paired RNA-Seq reads were aligned with STAR (Dobin et al., 2013) against the UCSC human reference genome (hg19). We performed RNA-seq differential expression analysis using the DESeq2 package with default parameters (Love et al., 2014). We use Gene Set Enrichment Analysis (GSEA) software to perform pathway analysis (Mootha et al., 2003; Subramanian et al., 2005). RNA-sequencing data are available via the GEO accession number: GSE208132.

### Statistical analysis

We performed statistical analysis using R Statistical Software (version 4.0.5; R Foundation for Statistical Computing, Vienna, Austria). We compared continuous variables using unpaired t-tests or Mann–Whitney U-tests (when the data sets were not normally distributed) between the relapse and non-relapse groups. We also applied p. adjust function in R (version 4.0.2) to estimate false discovery rate (FDR). An FDR-adjusted *p*-value of 0.05 indicates that 5% of significant tests will result in false positives (Ho et al., 2018; Ho et al., 2019; Ho et al., 2020). We analyzed categorical variables using a *x* ^2^ test or Fisher’s exact test. Data were presented as mean ± S.E.M. Figure 3 and Figure 5 were analyzed using one way ANOVA, followed by Tukey’s multiple comparison tests for individual comparisons when significant effects were detected (GraphPad Prism 8). *p* < 0.05 was considered statistically significant.

## Results

### Characteristics of study participants

We previously recruited 442 AUD patients to participate in our acamprosate clinical trial, and 267 of those 442 patients returned for the 3 months follow-up visit (Karpyak et al., 2014). The primary outcome was abstinence (Karpyak et al., 2014; Karpyak et al., 2019). We defined relapse (n = 110) as return to alcohol use during the 3 months of acamprosate treatment, and non-relapse (n = 157) as abstinence from alcohol (no alcohol use) during 3 months of acamprosate treatment. Nearly two-thirds of the patients were men, however, sex did not substantially influence acamprosate treatment response. Baseline alcohol craving scores, as determined by the PACS scale, were significantly higher in the relapse group than in the non-relapse group (*p* = 9.36E-06) (Table 1) (Ho et al., 2022b). The PACS is one of the most commonly used assessments for alcohol craving (Flannery et al., 1999; Ray et al., 2013). It is a five item self-report craving scale and each question is scaled from 0–6 (Flannery et al., 1999). A higher PACS score represents higher alcohol craving intensity. However, there is no established cutoff PACS score to determine the risk of relapse or the “severity” of craving. Furthermore, we observed that drinking patterns between the two groups appeared to differ, even though the number of total drinks prior to acamprosate treatment was similar between the relapse and non-relapse groups (Table 1).

**TABLE 1:** Demographic and clinical characteristics of the subjects.

	Relapse n = 110	Non-relapse n = 157	*p* value
	Mean±SD or n (%)	Mean±SD or n (%)	
Age (years)	41.59 ± 12.04	42.39 ± 11.58	NS
Sex: male	67 (60.9%)	112 (71.3%)	NS
Race: White	100 (90.9%)	147 (93.6%)	NS
Baseline PHQ9 Score	10.24 ± 6.14	8.79 ± 6.04	0.016
Baseline PACS Score	15.44 ± 8.47	11.91 ± 7.37	9.36E-06
Baseline GADS Score	9.35 ± 5.88	8.66 ± 5.80	NS
Alcohol consumption measure (TLFB 30 days prior to enrollment)			
Total drinks per month	118.30 ± 120.54	102.00 ± 123.26	NS
Number of drinking days	10.34 ± 8.83	7.98 ± 7.96	0.005
Number of heavy drinking days	9.15 ± 8.23	7.14 ± 7.60	0.010
Average drinks per drinking day	10.12 ± 8.62	9.60 ± 10.26	NS
Average drinks per drinking week	26.90 ± 27.36	23.45 ± 28.61	NS
Average drinks per drinking month	115.31 ± 117.25	100.51 ± 122.64	NS

PHQ-9, The Patient Health Questionnaire (PHQ)-9; PACS, The penn alcohol craving scale; GADS, General Anxiety Disorder-7; TLFB, timeline followback data. Relapse was defined as having one standard drink during 3 months of acamprosate treatment, while non-relapse was defined as the maintenance of abstinence from alcohol during 3 months of acamprosate treatment. SD: standard deviation. NS: non-significant.

### Plasma proteomics using the Olink target 96 inflammation panel and acamprosate treatment response

We used baseline plasma proteomics to identify potential markers associated with alcohol relapse risk and/or alcohol craving during 3 months of acamprosate treatment (Figure 1A). Acamprosate blood drug levels during the 3 months of follow-up visits were similar between the relapse group and the non-relapse group (Figure 1B). TNFSF10 was the most significant protein that was associated with relapse to alcohol use as well as relapse to heavy drinking during the 3 months of acamprosate treatment (Figures 1C,D, Supplementary Tables S2-S3). Specifically, TNFSF10 was significantly elevated in the non-relapse group as compared to the relapse group (Figures 1C,D). Furthermore, TNFSF10 was positively correlated with time until first drink and time until heavy drinking (Supplementary Tables S4-S5). Since baseline alcohol craving was associated with acamprosate treatment outcome (Table 1), we found that baseline TNFSF10 level was negatively correlated with baseline PACS (Figure 1E, and Supplementary Table S6). Although sex did not substantially influence acamprosate treatment response (Table 1), we observed a series of proteins that displayed sex-related differences in our AUD patients (Supplementary Table S7). We then determined whether associations between the inflammatory markers and acamprosate treatment outcomes differed significantly between men and women, and we observed no significant interactions.

### Alcohol craving and alcohol relapse risk

**FIGURE 2:**
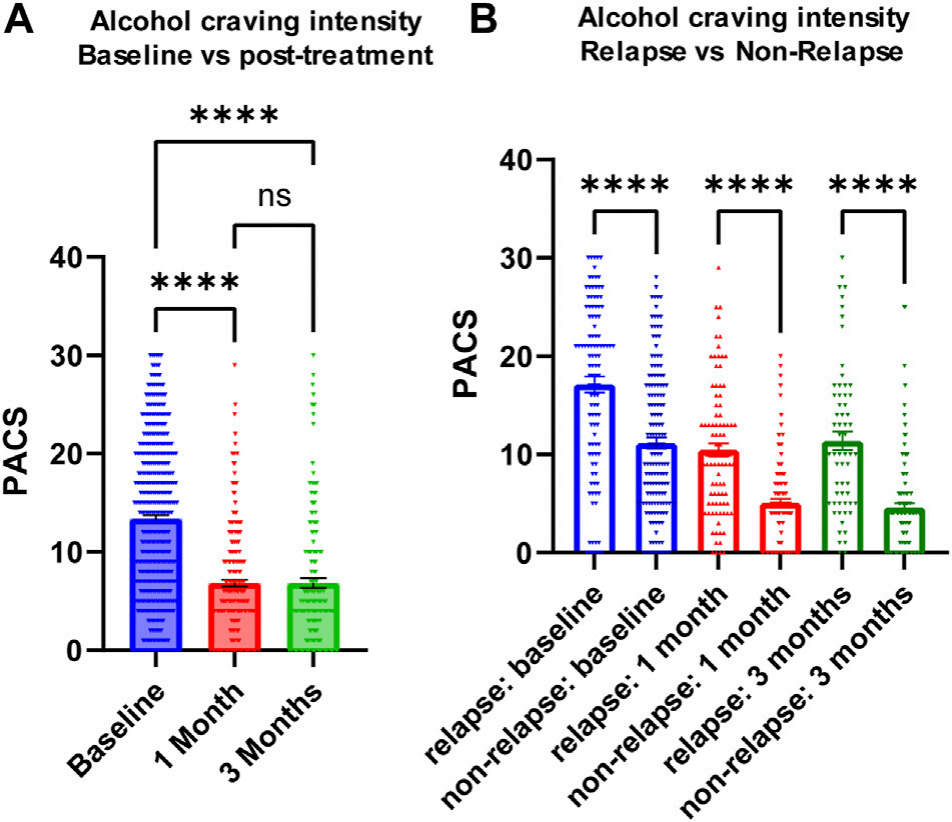
Alcohol craving predicts acamprosate treatment response. **(A)** PACS scores before and after acamprosate treatment for participants in the acamprosate trial (*F*_(2,923)_ = 97.81, *p* < 0.0001). **(B)** PACS scores were significantly higher in the relapse group than in the non-relapse group at baseline, 1 month and 3 months after acamprosate treatment (*F*_(5,685)_ = 59.73, *p* < 0.0001). ****p < 0.0001. Relapse was defined as taking one drink during 3 months of acamprosate treatment, while non-relapse was defined as remaining abstinent during 3 months of acamprosate treatment.

We measured craving intensity using PACS at baseline as well as 1 month and 3 months after the initiation of acamprosate therapy. As anticipated, the average PACS scores declined significantly after acamprosate treatment (Figure 2A). Craving scores were significantly higher in the relapse group (return to drink) as compared to the non-relapse group (sobriety) (Figure 2B). This trend remained during acamprosate treatment, although both groups showed significant reductions in PACS scores after acamprosate treatment (Figure 2B).

### TNFSF10 was associated with acamprosate treatment outcome

**FIGURE 3:**
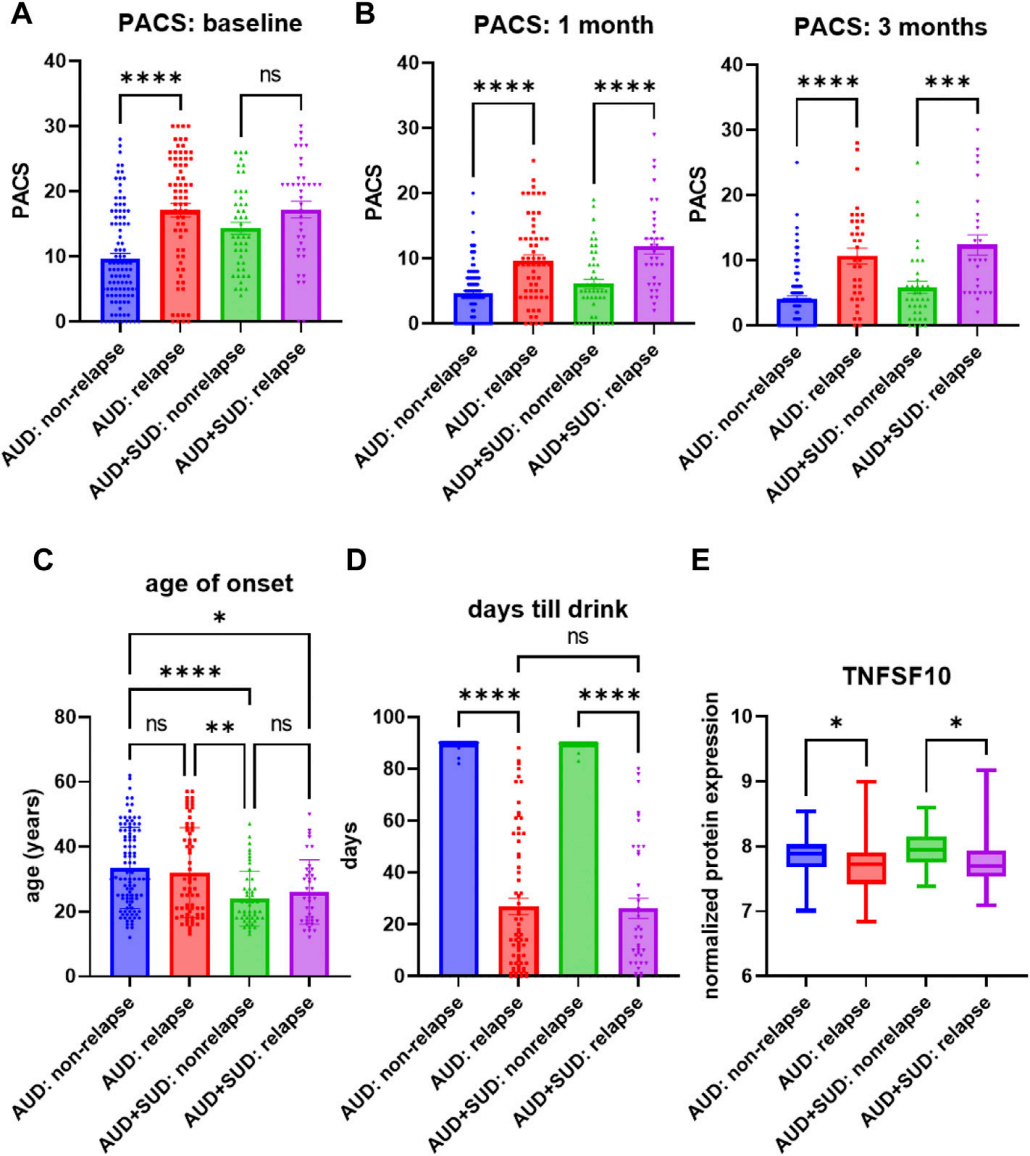
Craving intensity and acamprosate treatment. **(A)** Baseline PACS was associated with acamprosate treatment response only in patients with alcohol use disorder but not AUD patients with a history of other substance use disorders (SUD), (*F*_*(3, 252*)_ = 15.76, *p* < 0.0001). Relapse was defined as taking one drink during 3 months of acamprosate treatment, while non-relapse was defined as remaining abstinent during 3 months of acamprosate treatment. **(B)** Higher PASC was observed in the relapse groups after acamprosate treatment for 1 month, (*F*_*(3, 232*)_ = 20.85, *p* < 0.0001), and 3 months, (*F*_*(3, 186)*_ = 19.62, *p* < 0.0001). **(C)** Age of onset for AUD was significantly lower in AUD subjects with history of other SUD (*F*_*(3, 251*)_ = 8.808, *p* < 0.0001). **(D)** The number of days until first alcohol use during the 3 months of acamprosate treatment was similar between AUD patients with and without a history of SUD, (*F*_*(3, 254*)_ = 303.7, *p* < 0.0001). **(E)** Lower baseline plasma TNFSF10 level was observed in the relapse groups regardless of a history of other SUD, (*F*_*(3, 232*)_ = 6.376, *p* < 0.0001). ****p < 0.0001, ***p < 0.001, **p < 0.005, *p < 0.05.

Similar to the majority of studies on substance use disorders, our acamprosate trial was designed to study individuals with AUD, and multiple concurrent substance use was considered an exclusion criterion. However, approximately one third of our AUD patients had a history of other substance use disorders (SUD), i.e., cannabis use disorder or cocaine use disorder (Supplementary Table S8) (Karpyak et al., 2019). Of importance, AUD patients who were without a history of other SUD and who relapsed to alcohol use during the 3 months of acamprosate treatment showed higher baseline PACS, as compared to AUD patients without a history of other SUD and who maintained sobriety during the 3 months of acamprosate treatment (Figure 3A). However, similar baseline PACS values were observed in AUD patients with a history of other SUD regardless of acamprosate treatment outcomes (Figure 3A). As expected, all four groups showed significant reductions in PACS in response to acamprosate treatment (Supplementary Figure S1), and the relapse groups displayed higher PACS values than the non-relapse groups after acamprosate treatment (Figure 3B). The age of onset for AUD was significantly lower in AUD subjects with a history of other SUD but it was not associated with acamprosate treatment response (Figure 3C). No significant difference was observed between AUD with or without a history of other SUD with regard to the number of days until the first drink during the 3 months of acamprosate treatment (Figure 3D). These findings suggest that a history of SUD might not have implications for acamprosate treatment response. Consistently, baseline plasma TNFSF10 levels were significantly associated with acamprosate treatment outcomes for AUD patients with and without a history of other SUD (Figure 3E). In summary, this series of observations demonstrate that TNFSF10 levels, at least in the present study, were associated with acamprosate treatment outcomes, thus raising the question of the underlying biology that might drive the differences in TNFSF10 that we observed, a question that we began to address in the studies described subsequently.

### TNFSF10 mRNA expression and acamprosate treatment response

**FIGURE 4:**
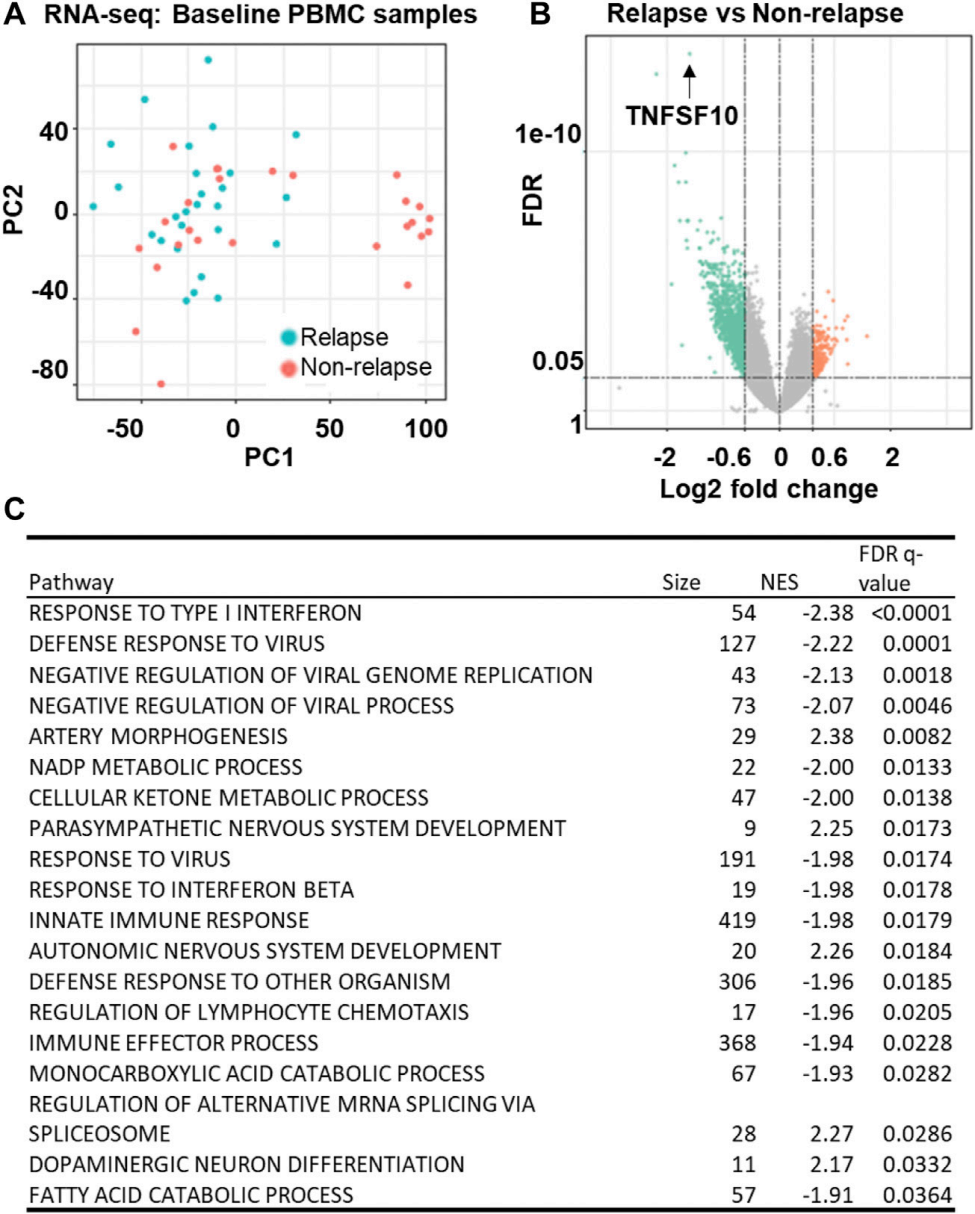
Gene expression profiles in baseline PBMC sample from AUD patients. **(A)** Principal components analysis (PCA) of gene expression profiles in PBMCs obtained at baseline from AUD patients (relapse: n = 27, non-relapse: n = 26). Relapse was defined as taking one drink during 3 months of acamprosate treatment, while non-relapse was defined as remaining abstinent during 3 months of acamprosate treatment. **(B)** A volcano plot showing expression profiles for the most differentially expressed genes between the relapse and non-relapse groups based on the PBMC RNA-seq data (FDR<0.05). **(C)** Pathway analysis of the PBMC RNA-seq data was performed using gene set enrichment analysis (GSEA) software (Mootha et al., 2003; Subramanian et al., 2005). NES is the normalized enrichment score to account for the size of each gene set.

We began this series of studies by determining genome-wide gene expression profiles for peripheral blood mononuclear cells (PBMCs) obtained from a subset of AUD patients who gave baseline blood sample for banking (relapse: n = 27, non-relapse: n = 26) (Figure 4A). Strikingly, TNFSF10 was the top differentially expressed gene (*p* = 1.67E-14) (Figure 4B and Supplementary Table S9). Specifically, TNFSF10 mRNA expression in PBMC was significantly lower in the relapse group. In addition, pathway analysis of those genome-wide expression data focused on immune-related pathways including interferon response signalling pathways (Figure 4C and Supplementary Table S10). This series of observations demonstrated that TNFSF10 was associated with acamprosate treatment response, thus raising the question of whether acamprosate might regulate TNFSF10 concentrations.

### Acamprosate regulates TNFSF10 concentrations

We next set out to determine whether TNFSF10 concentrations could be regulated by acamprosate using LCLs derived from AUD patients for whom we knew their acamprosate treatment response. LCLs are EB virus transformed B cells and this cell model has been utilized repeatedly to generate and test pharmacogenomic hypotheses and has proven to be a powerful research tool for functional genomic studies (Ho et al., 2016; Ho et al., 2017; Ho et al., 2018). We should also point out that TNFSF10 is expressed mostly in immune cells. therefore, LCLs are a practical model for the functional assay described subsequently.

**FIGURE 5:**
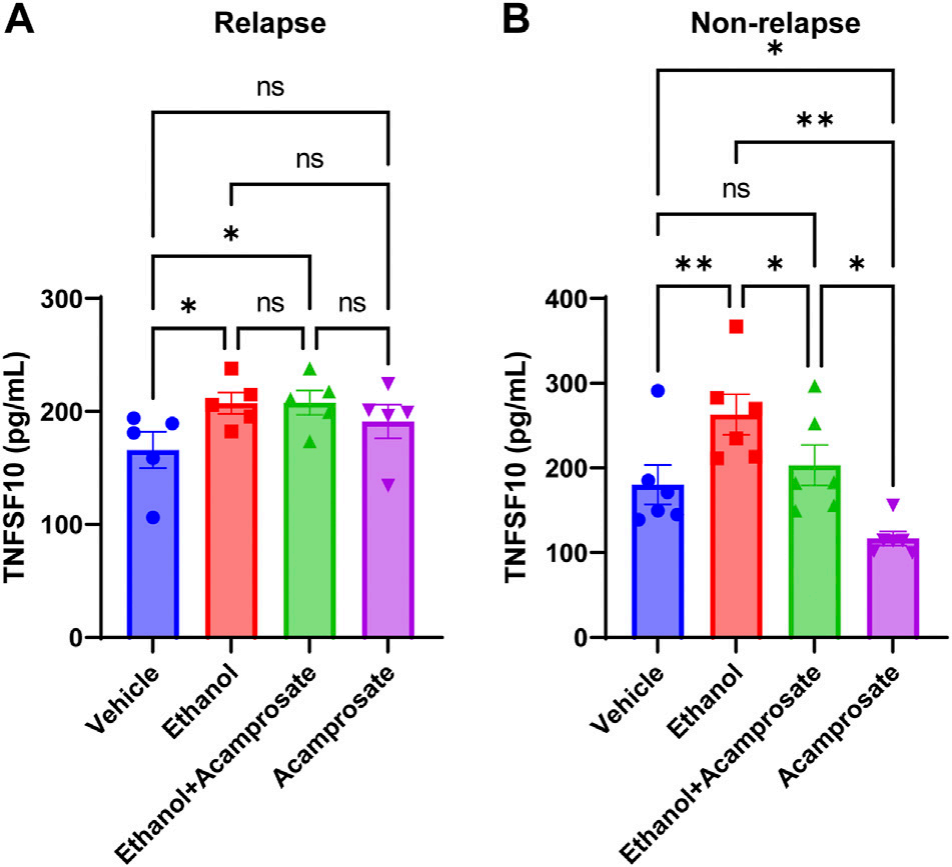
TNFSF10 concentrations were measured with and without drug exposure in LCLs derived from AUD patients with known acamprosate treatment outcomes. **(A)** Ethanol could induce TNFSF10 concentrations in LCLs derived from AUD patients who relapsed during 3 months of acamprosate treatment. However, acamprosate did not influence TNFSF10 concentrations (*F*_(4, 12)_ = 6.420, *p* = 0.005). ELISA was performed in AUD patient-derived lymphoblastoid cell lines (LCLs). TNFSF10 concentrations were determined with and without treatment with EtOH (25 mM), or acamprosate (5µM) for 7 days. *A *p* value ≤ 0.05 was considered statistically significant. **(B)** Ethanol could induce TNFSF10 concentrations in LCLs derived from AUD patients who remained abstinent during 3 months of acamprosate treatment. However, acamprosate could decrease the levels of TNFSF10 (*F*_(5, 15)_ = 12.76, *p* < 0.0001). **p < 0.005, *p < 0.05. Three independent experiments were performed. All values are mean±S.E.M.

TNFSF10 concentrations in LCLs could be induced by ethanol in both groups (Figures 5A,B). However, TNFSF10 concentrations were significantly decreased in LCLs incubated in the presence of acamprosate in the non-relapse group, whereas the effect was not significant in the relapse group (Figure 5). Furthermore, incubation with ethanol significantly induced TNFSF10 levels. However, those effects could be reversed by acamprosate only in AUD patients who responded to acamprosate treatment (Figure 5). These findings further strengthen the conclusion that TNFSF10 might play a role in acamprosate treatment response.

## Discussion

AUD is the most common SUD worldwide (Suen et al., 2021). Similar to most psychiatric disorders, AUD is diagnosed based on a list of clinical symptoms included in the Diagnostic and Statistical Manual for Mental Disorder, 5^th^ edition, rather than through the use of biologically based biomarkers. The present study was designed to use proteomics data as a quantitative biological trait as a step toward identifying inflammatory modulators that might be associated with acamprosate treatment outcomes. Previous studies linked elevated craving intensity to an increased probability of alcohol relapse among AUD patients (Haass-Koffler et al., 2014). Our present study demonstrates that baseline craving intensity appeared to be the most significant clinical phenotype for alcohol relapse during 3 months of acamprosate treatment (Table 1). Acamprosate is an anti-craving drug. As expected, there was a significant reduction in alcohol craving intensity in patients with AUD after acamprosate treatment (Figure 2A). However, patients who relapsed to alcohol use appeared to have higher alcohol craving intensity over time throughout the study (Figure 2B).

We found that TNFSF10 levels were associated with alcohol relapse risk and with baseline alcohol craving intensity although, obviously, those observations require replication (Figures 1C–E). It should be pointed out that the limitation of these correlational data is that they do not address causality between changes in peripheral inflammation and modifications in craving and/or alcohol consumption. TNFSF10, also known as TRAIL, belongs to the tumor necrosis factor (TNF) ligand family. A recent study reported that TNFSF10 is ethanol inducible, and that the inhibition of TNFSF10 blocked ethanol-induced cell death via TLR7 (Qin et al., 2021). In parallel, our functional genomic study showed that TNFSF10 could be induced by ethanol in LCLs derived from AUD patients. Strikingly, these effects were reversed by acamprosate only in patients who responded to acamprosate treatment (Figure 5). We should point out that we do not have plasma samples after acamprosate treatment, because our acamprosate clinical trial was not originally designed to study peripheral omics-based biomarkers, but rather genomic biomarkers associated with acamprosate treatment response (Karpyak et al., 2014; Biernacka et al., 2021). Therefore, baseline blood samples were collected as the source of DNA, and baseline plasma samples were stored for future studies such as those reported here. Despite these limitations, our work represents an important contribution by providing novel insight into individual variation in acamprosate treatment response. To our knowledge, there are no prior studies addressing the biological roles of these inflammatory modulators in AUD disease risk and/or acamprosate treatment outcome. Further investigation is warranted and needed to explore mechanisms involved in TNFSF10 and its relationship with variation in acamprosate response phenotypes.

In conclusion, we have compared proteomic profiles focusing on inflammatory modulators between patients who maintained sobriety and those who relapsed based on “any” alcohol use during 3 months of acamprosate treatment. This study identified baseline plasma TNFSF10 concentration as associated with variation in acamprosate treatment outcomes among AUD patients. These observations also suggest that the application of omics-based approaches may be a helpful approach for identifying biologic markers that could potentially predict acamprosate treatment response and alcohol craving intensity. As a result, these observations represent an important step toward advancing our understanding of the pathophysiology of AUD and molecular mechanisms associated with acamprosate treatment response.

## Data Availability

The datasets presented in this study can be found in online repositories. The names of the repository/repositories and accession number(s) can be found below: https://www.ncbi.nlm.nih.gov/geo/, GSE208132.
