# Supplementary figures and images for: Plasma TNFSF10 levels associated with acamprosate treatment response in patients with alcohol use disorder
Source: Front Pharmacol. 2022 Sep 1;13:986238. doi: 10.3389/fphar.2022.986238 (PMC9475292; doi:10.3389/fphar.2022.986238)

## Slide 1
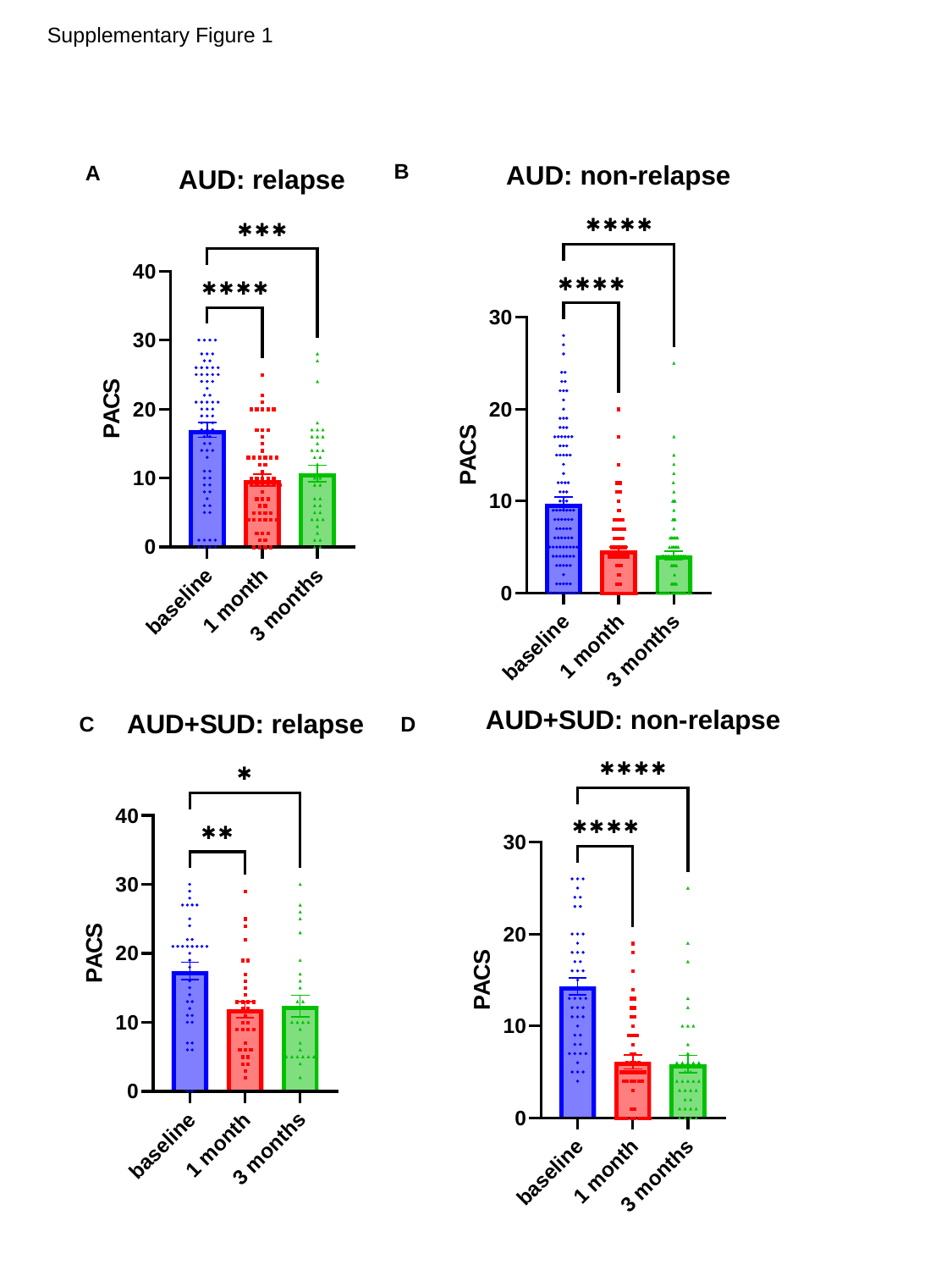

Supplementary Figure 1
B
A
D
C

Supplement: Supplementary file 1 [file Presentation1.PPTX]
