# Supplementary material for: Plasma TNFSF10 levels associated with acamprosate treatment response in patients with alcohol use disorder
Source: Front Pharmacol. 2022 Sep 1;13:986238. doi: 10.3389/fphar.2022.986238 (PMC9475292; doi:10.3389/fphar.2022.986238)
Supplement: Supplementary file 2 [file DataSheet1.docx]

Supplementary Material

Plasma TNFSF10 levels associated with acamprosate treatment response in patients with alcohol use disorder

# Ming-Fen Ho, Ph.D.^1*^, Cheng Zhang, Ph.D.^1^, Irene Moon, M.S^1^, Brandon J. Coombes, Ph.D.^2^, Joanna Biernacka, Ph.D.^2^, Michelle Skime, M.S.^3^, Doo-Sup Choi, Ph.D.^1^, Paul E. Croarkin, D.O, M.S., Mark A. Frye, M.D.^3^, Quyen Ngo, Ph.D.^4^, Cedric Skillon, M.D.^4^, Tyler S. Oesterle, MD. M.P.H.^3^, Victor M Karpyak, MD, Ph.D^3^, Hu Li, Ph.D.^1^, and Richard M. Weinshilboum, M.D.^1*^

^1^ Department of Molecular Pharmacology and Experimental Therapeutics; Mayo Clinic; Rochester, Minnesota, USA

^2^ Division of Computational Biology, Quantitative Health Sciences; Mayo Clinic; Rochester, Minnesota, USA

^3^ Department of Psychiatry and Psychology, Mayo Clinic; Rochester, Minnesota, USA

^4^ Hazelden Betty Ford Foundation; Center City, Minnesota, USA

*** Correspondence:**

Ming-Fen Ho, Ph.D., Mayo Clinic 200 First Street SW, Rochester, MN 55905,

Phone: 507-284-2790, Email: ho.mingfen@mayo.edu

Richard Weinshilboum, MD, Mayo Clinic 200 First Street SW, Rochester, MN 55905,

Phone: 507-284-2790, Email: [weinshilboum.richard@mayo.edu](mailto:weinshilboum.richard@mayo.edu)

**Supplementary Figure 1.** PACS was reduced in after acamprosate treatment. **(A)** PACS in the relapse group of AUD patients without a history of SUD, (*F _(2, 164)_* = 16.18, p<0.0001). **(B)** PACS in the non-relapse group of AUD patients without a history of SUD, **(***F _(2, 291)_* = 31.6**,** p<0.0001). **(C)** PACS in the relapse group of AUD patients with a history of SUD, (*F _(2, 96)_* = 5.991, p=0.003). **(D)** PACS in the non-relapse group in AUD patients with a history of SUD, (*F _(2, 128_)* = 31.95, p<0.0001). Relapse was defined as taking one drink during three months of acamprosate treatment, while non-relapse was defined as remaining abstinent during three months of acamprosate treatment.

**Supplementary Table 1.** Olink Proteomics inflammatory panel and missing data frequency.

**Supplementary Table 2.** Plasma concentrations of inflammatory markers in relapse and non-relapse groups. Relapse was defined as taking one drink during three months of acamprosate treatment, while non-relapse was defined as remaining abstinent during three months of acamprosate treatment.

**Supplementary Table 3.** Plasma concentrations of inflammatory markers in heavy relapse and non-heavy relapse groups. Heavy relapse was defined as four or more standard drinks per day for a woman and five or more standard drinks per day for a man.

**Supplementary Table 4.** Correlation of time till relapse and baseline inflammatory markers. Number of sober days was included as a covariate.

**Supplementary Table 5.** Correlation of time till heavy relapse and baseline inflammatory markers. Number of sober days was included as a covariate.

**Supplementary Table 6.** Correlation of baseline PACS and plasma proteomics. Number of sober days prior to enrollment was included as a covariate.

**Supplementary Table 7,** Sex-specific differences in protein concentrations.

**Supplementary Table 8.** History of substance dependence for the 442 AUD subjects enrolled in the acamprosate clinical trial.

**Supplementary Table 9.** RNA-seq in PBMC from AUD patients. Relapse: n=27, non-relapse: n=26. Relapse was defined as taking one drink during three months of acamprosate treatment, while non-relapse was defined as remaining abstinent during three months of acamprosate treatment.

**Supplementary Table 10.** Pathway analysis using RNA-seq data in PBMC from AUD patients. Relapse: n=27, non-relapse: n=26. Relapse was defined as taking one drink during three months of acamprosate treatment, while non-relapse was defined as remaining abstinent during three months of acamprosate treatment.
